# Targeting IGF2BP3 in Cancer

**DOI:** 10.3390/ijms24119423

**Published:** 2023-05-29

**Authors:** Xin Liu, Jiayu Chen, Wenliang Chen, Yangtao Xu, Yang Shen, Ximing Xu

**Affiliations:** Cancer Center, Renmin Hospital of Wuhan University, Wuhan 430060, China

**Keywords:** IGF2BP3, m6A binding protein, cancer-promoting factor, radiosensitivity, tumor immunity

## Abstract

RNA-binding proteins (RBPs) can regulate multiple pathways by binding to RNAs, playing a variety of functions, such as localization, stability, and immunity. In recent years, with the development of technology, researchers have discovered that RBPs play a key role in the N6-methyladenosine (m6A) modification process. M6A methylation is the most abundant form of RNA modification in eukaryotes, which is defined as methylation on the sixth N atom of adenine in RNA. Insulin-like growth factor 2 mRNA-binding protein 3 (IGF2BP3) is one of the components of m6A binding proteins, which plays an important role in decoding m6A marks and performing various biological functions. IGF2BP3 is abnormally expressed in many human cancers, often associated with poor prognosis. Here, we summarize the physiological role of IGF2BP3 in organisms and describe its role and mechanism in tumors. These data suggest that IGF2BP3 may be a valuable therapeutic target and prognostic marker in the future.

## 1. Introduction

RNA-binding proteins (RBPs) are proteins that bind to single- or double-stranded RNA in cells, which together form ribonucleoprotein complexes [1]. RBPs play a role in signal transduction through RNA editing, RNA splicing, RNA polyadenylation, or RNA degradation [2]. In recent years, research on RBPs in tumors has been increasing. Studies have shown that RBPs play a key role in the N6-methyladenosine (m6A) modification process. M6A methylation is the most prevalent mRNA modification in eukaryotes and is involved in cell differentiation, tissue development, and tumorigenesis [3,4,5]. With the deepening of research and the emergence of high-throughput sequencing technology, researchers’ understanding of m6A methylation has gradually increased. M6A modification is in not only mRNA but also various non-coding RNAs [6,7].

M6A modification is installed by methyltransferases (m6A writers) and possibly removed by demethylases (m6A erasers); this process is recognized by binding proteins (m6A readers) [8]. The m6A methyltransferases mainly include methyltransferase-like protein 3 (METTL3), methyltransferase-like protein 14 (METTL14), methyltransferase-like 16 (METTL16), Wilms tumor 1-associated protein (WTAP), RNA-binding motif protein 15 (RBM15), and zinc-finger CCCH-type-containing 13 (ZC3H13) [9]. M6A demethylases include α-ketoglutarate-dependent dioxygenase alk B homolog 5 (ALKBH5) and fat mass and obesity-associated protein (FTO) [10]. In addition, the m6A-binding proteins consist of YT521-B homology domain family proteins (YTHDF1/2/3), YT521-B homology domain containing 1 and 2 (YTHDC1/2), eukaryotic translation initiation factor 3 (eIF3), insulin-like growth factor 2 mRNA-binding proteins (IGF2BP1/2/3), and heterogeneous nuclear ribonucleoproteins (HNRNPA2/B1 and HNRNPC/G) [11,12,13].

*IGF2BP3* was initially identified as a highly expressed gene in pancreatic cancer, and the encoded protein was then confirmed to be an m6A reader [13,14]. IGF2BP3 consists of two RNA recognition motif (RRM) domains and four K homology (KH) domains [15]. IGF2BP3 recognizes the m6A site through the KH domains, thereby promoting RNA stability and translation efficiency in an m6A-dependent manner [16]. In addition, IGF2BP3 has a variety of physiological functions, including promoting the embryonic development of organisms, regulating fetal megakaryocytes, and inducing fetal hemoglobin in adult erythroblasts. The expression of IGF2BP3 is increased in multiple tumors, suggesting that IGF2BP3 plays the role of a tumor-promoting factor. In this review, we describe the physiological functions of IGF2BP3. Then, we outline the role and specific mechanisms of IGF2BP3 in tumors in either m6A-dependent or m6A-independent manners to provide new insights for the treatment of malignant tumors.

## 2. Physiological Roles of IGF2BP3

IGF2BP3 plays an important role in the embryonic development of organisms. IGF2BP3 was expressed during mouse embryonic development and peaked at day 12.5, followed by a gradual decline [17,18]. IGF2BP3 is mainly expressed in the brain, nose, gill arch, viscera, vertebrae, and skin [19]. Mori et al. found that IGF2BP3 expression is associated with neuronal differentiation in mouse embryonic stages [20]. In developmental studies of Xenopus laevis, loss of the Vg1 RBP, which is homologous to human IGF2BP3, resulted in abnormal lens and neural tube formation and loss of the dorsal fin [21]. In zebrafish, Igf2bp3 is essential for embryonic development and can maintain maternal RNA stability [22,23]. Although research is lacking on the role of IGF2BP3 in human development, IGF2BP3 is known to be involved in the development of fetal megakaryocytes and the induction of fetal hemoglobin in adult erythroblasts [24,25]. IGF2BP3 is mainly expressed in reproductive tissues in adult organs, and future studies can focus on the role of IGF2BP3 in reproductive function [26].

Therefore, IGF2BP3 is an important regulator of developmental processes in organisms. Embryonic development is a precisely regulated process, and IGF2BP3 appears and declines at specific times and locations, suggesting that IGF2BP3 is an important player in normal embryonic development.

## 3. IGF2BP3 in Different Human Cancers

### 3.1. Leukemia

Leukemia is one of the most common malignant tumors of the blood system, and acute myelogenous leukemia (AML) accounts for nearly one-third of all diagnosed leukemias [27].

Zhang et al. demonstrated that IGF2BP3 levels were significantly elevated in bone marrow samples from AML patients and were associated with a poor prognosis. IGF2BP3 enhances the stability of *RCC2* mRNA by reading the m6A modification sites, thereby reducing AML cell apoptosis and promoting the progression of acute myeloid leukemia [28]. Ko et al. found that IL-18 plays an anti-apoptotic role in acute myeloid leukemia by promoting the interaction between IGF2BP3 and HuR and contributing to the stabilization of *COX-2* mRNA [29].

Cytarabine (Ara-C), in combination with anthracyclines, is the standard treatment for patients with AML [30]. However, drug resistance is a major obstacle in the treatment of AML patients. Gou et al. found that apoptotic bodies carrying IGF2BP3 were phagocytized by recipient cells, thereby activating the PI3K–AKT pathway and enhancing the ability of recipient cells to escape apoptosis during chemotherapy [31]. This suggested that IGF2BP3 is a target for reversing cytarabine resistance.

In addition, IGF2BP3 plays a role in MLL-rearranged leukemia. The deletion of *Igf2bp3* significantly increased mouse survival and reduced the number and self-renewal capacity of leukemia-initiating cells [32].

**Figure 1:**
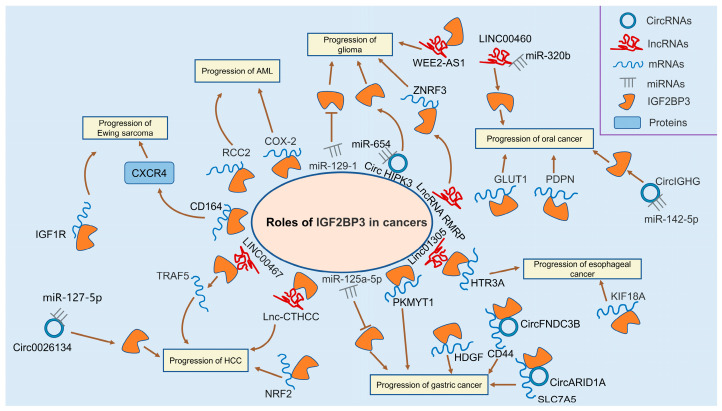
Roles of IGF2BP3 in acute myelogenous leukemia, glioma, Ewing sarcoma, hepatocellular carcinoma, gastric cancer, oral cancer, and esophageal cancer.

Although the function of IGF2BP3 in other types of hematological malignancies is unclear, a promoting role of IGF2BP3 in leukemia has been identified (Figure 1).

### 3.2. Glioma

Central nervous system tumors account for a large proportion of the morbidity and mortality in the United States; from 2008 to 2017, the incidence of malignant brain tumors among children and adolescents increased by 0.5% to 0.7% per year [33].

Glioma is a common nervous system tumor. Chen et al. demonstrated that the expression of IGF2BP3 was significantly increased in glioma compared with that in normal tissue [34]. Kouhkan et al. found that microRNA-129-1 negatively regulates IGF2BP3 to inhibit glioma cell proliferation and induce cell cycle arrest [35]. Jin et al. confirmed that Circ HIPK3 promotes the proliferation and invasion of glioma cells by interacting with miR-654 and upregulating the expression of IGF2BP3 [36]. Nomura et al. showed that genes, including *IGF2BP3*, are upregulated due to the demethylation of the promoter [37]. This suggests that future studies on glioma should further focus on the relationship between methylation and *IGF2BP3* gene expression.

Temozolomide is an approved first-line treatment for newly diagnosed glioblastoma [38]. However, most patients eventually develop resistance to temozolomide. Liu et al. found that lncRNA RMRP regulates *ZNRF3* mRNA stability through an RNA-induced silencing complex (RISC)-dependent mechanism mediated by IGF2BP3, leading to temozolomide resistance in glioma cells [39].

Dasatinib is an approved CNS penetrant for glioma [40,41]. Li et al. found that IGF2BP3 enhances the stability of lncRNA WEE2-AS1 in an m6A-dependent manner to promote GBM progression and attenuate the therapeutic sensitivity of dasatinib in glioma cells. Moreover, site 1 in exon 7 of lncRNA WEE2-AS1 is the main locus of m6A regulation [42]. These findings indicated that IGF2BP3 is a promising target for the treatment of drug resistance in glioma.

Tumor-associated macrophages (TAMs) are progression-promoting infiltrating immune cell types in glioma that are capable of shaping the immunosuppressive tumor microenvironment [43,44]. Pan et al. demonstrated that circNEIL3 stabilized IGF2BP3 protein by preventing HECTD4-mediated ubiquitination from promoting glioma progression and macrophage immunosuppressive polarization [45]. This study further confirmed the tumor-promoting role of IGF2BP3 in glioma. The interaction between tumor cells and the tumor microenvironment plays an important role in tumor progression and the treatment response. However, studies on the role of IGF2BP3 in the tumor microenvironment are scarce. Researchers should focus more on the close relationship between IGF2BP3 and the tumor microenvironment.

Johnson et al. identified a new signature for predicting OS in glioma. This model was able to maintain the prognostic value after adjustment for several clinical variables [46]. Other researchers may try to use this signature to verify its efficacy.

In conclusion, IGF2BP3 acts as a tumor-promoting factor in glioma, and targeting its upstream regulators may be an approach to inhibit glioma progression. In addition, the regulatory mechanism of IGF2BP3 in the tumor microenvironment remains to be further studied (Figure 1).

### 3.3. Meningioma

Meningioma is also a common primary intracranial tumor, some of which are highly aggressive and have a low survival rate [47].

Predicting meningioma recurrence is one of the major clinical challenges. Hao et al. found that the 5-year recurrence-free rate and overall survival rate of IGF2BP3-positive patients were much lower than those of IGF2BP3-negative patients, confirming that IGF2BP3 is a potential independent prognostic biomarker of the high-risk recurrence of meningioma [48].

Overall, IGF2BP3 has been less studied in meningioma and may be a suitable predictor of recurrence in meningioma patients. In addition, IGF2BP3 can be explored in combination with other predictors to achieve higher-accuracy prediction results.

### 3.4. Ewing Sarcoma

Ewing sarcoma (ES) is a common malignant bone tumor in children and young adults, characterized by rapid growth and early metastasis [49].

In 2018, a study found that IGF2BP3 is a tumor-promoting factor in Ewing sarcoma. In addition, *ABCF1* mRNA acts as a sponge, partially inhibiting IGF2BP3 functioning [50]. Mancarella et al. confirmed that IGF2BP3 regulates the expression of CXCR4 through CD164, thereby promoting the migration of ES cells, and IGF2BP3 was found to be an effective indicator for predicting the recurrence of patients [51]. IGF2BP3 bound and stabilized *IGF1R* mRNA and actively regulated its function, thereby promoting ES cell migration and growth [52].

IGF2BP3 is expected to be one of the targets to inhibit the metastasis of Ewing sarcoma, but more research evidence is needed to support this (Figure 1).

### 3.5. Hepatocellular Carcinoma

Hepatocellular carcinoma (HCC) is a major contributor to the world’s cancer burden and the third most common cause of cancer-related death [53].

Many researchers have focused on the function of IGF2BP3 in HCC. Jeng et al. found that the expression of IGF2BP3 was increased in HCC patient samples, which was correlated with alpha-fetoprotein, tumor grade, tumor stage, and metastasis, and that IGF2BP3 depletion could reduce HCC cell motility and invasion [54]. Jiang et al. found that LINC00467 interacts with IGF2BP3 to enhance the mRNA stability of *TRAF5*, thereby promoting the proliferation and metastasis of HCC cells [55]. Xia et al. found that IGF2BP3 increases the stability of lnc-CTHCC in HCC cells by recognizing m6A modification, thus promoting proliferation and invasion [56]. Zhang et al. found that circ0026134 upregulates the expression of IGF2BP3 through the sponge miR-127-5p, thereby promoting the proliferation and invasion of liver cancer cells [57]. Nguyen et al. found that the deletion of *Lin28b* in murine models reduces the liver tumor burden and prolongs survival, and *Igf2bp3* functions as a downstream gene of Lin28b [58].

The above studies show that IGF2BP3 plays a role in promoting cancer in HCC, and non-coding RNA participates in the progression of HCC by regulating the expression of IGF2BP3. Therefore, targeting these non-coding RNAs and reducing IGF2BP3 levels may be a strategy to delay the progression of HCC. Shaalan et al. demonstrated that Tamarix articulata and quercetin target the miR-1275/IGF2BP3 axis in liver cancer, thereby delaying tumor progression [59]. This study indicated the possibility of targeting non-coding RNAs to regulate IGF2BP3 expression in HCC.

Sorafenib is a systemic drug approved for hepatocellular carcinoma [60]. However, resistance to sorafenib is becoming more common. Li et al. found that an isocorydine derivative (d-ICD) negatively regulates the proliferation of CD133(+) cancer stem cells (CSCs) by targeting IGF2BP3, thereby inhibiting sorafenib resistance in HCC cells [61]. Lu et al. confirmed that IGF2BP3 inhibits ferroptosis in liver cancer cells by promoting the stability of *NRF2* mRNA in an m6A-dependent manner, resulting in a reduced therapeutic effect of sorafenib on nude mice. Furthermore, the +1482 base of NRF2 mRNA may be the binding site for IGF2BP3 [62]. These studies suggest that IGF2BP3 may be a target for reversing sorafenib resistance.

Various prognostic models of HCC also have the appearance of IGF2BP3. Li et al. used the TCGA database to construct a prediction model containing nine m6A/m1A/m5C- regulated genes (*YBX1*, *ZC3H13*, *YTHDF1*, *TRMT10C*, *YTHDF2*, *RRP8*, *TRMT6*, *LRPPRC*, and *IGF2BP3*). This risk model is related to tumor grade, clinical stage, T stage, and distant metastasis in HCC patients [63]. Zhang et al. screened and constructed immune-related gene prediction models. The model consisted of three immune genes (*G6PD*, *ZNF239*, and *IGF2BP3*), and patients in the high-risk group exhibited resistance to certain chemotherapy drugs [64]. Wang et al. screened out 10 central genes (*BIRC5*, *FOXM1*, *CENPA*, *KIF4A*, *DTYMK*, *PRC1*, *IGF2BP3*, *KIF2C*, *TRIP13*, and *TPX2*) related to immune cell infiltration. The expression of these central genes was higher in patients with clinically advanced HCC [65].

In conclusion, IGF2BP3 acts as a tumor-promoting factor in liver cancer and is involved in resistance to therapeutic drugs. In addition, IGF2BP3 participates in the construction of various prognostic models, indicating its ability to predict prognosis. Notably, the clinical predictive ability of IGF2BP3 also needs further verification (Figure 1).

### 3.6. Gastric Cancer

Gastric cancer (GC) is a common tumor disease, with more than 1 million people worldwide diagnosed with gastric cancer every year [66].

Zhang et al. found that microR-125a-5p can target IGF2BP3 and inhibit the proliferation of gastric cancer cells [67]. Hu et al. found that PKMYT1 is highly expressed in GC in both the tissue microarray and TCGA database. Additionally, IGF2BP3 helps to stabilize the mRNA stability of *PKMYT1* via its m6A modification site to promote GC cell invasion and migration [68]. Wang et al. confirmed that METTL3 in gastric cancer stimulates the m6A modification of *HDGF* mRNA, and then the m6A reader IGF2BP3 directly recognizes and binds to the m6A site on *HDGF* mRNA and enhances its stability. The METTL3/IGF2BP3/*HDGF* axis promotes tumor angiogenesis, tumor growth, and liver metastasis [69]. Ma et al. found that the expression of IGF2BP3 increases in gastric cancer tissue samples and cell lines. CircARID1A binds to IGF2BP3 and forms a circARID1A–IGF2BP3–*SLC7A5* RNA–protein ternary complex to regulate the AKT/mTOR pathway, thereby promoting GC proliferation [70]. Yu et al. identified that circ-TNPO3 was downregulated and IGF2BP3 and MYC were upregulated in GC samples. Circ-TNPO3 can act as a protein decoy for IGF2BP3, weakening the role of IGF2BP3 in stabilizing *MYC* mRNA, thereby inhibiting gastric cancer cell migration and proliferation. Furthermore, the 3′UTR of *MYC* is the direct location for binding to IGF2BP3 [71]. Hong et al. confirmed that circFNDC3B increases the level of CD44 associated with cell adhesion by forming a ternary complex of circFNDC3B–IGF2BP3–*CD44* mRNA [72]. Zhou et al. found that miR-34a is downregulated in gastric cancer and negatively correlates with IGF2BP3 expression. miR-34a inhibits proliferation and invasion and induces apoptosis through the miR-34a/IGF2BP3 axis in gastric cancer cells [73]. Ishii et al. confirmed that the knockout of *H19* in gastric cancer cells reduces the expression of IGF2BP3, MYC, ZEB1, and Snail1, thereby attenuating tumor cell proliferation and invasion [74].

Linitis plastica is a distinct type of gastric cancer with a poor prognosis. Liu et al. reported that IGF2BP3 is significantly upregulated in the linitis plastica compared with normal tissues and non-diffuse gastric cancer tissues and is associated with PI3K–AKT pathway activation [75].

In addition, Damasceno et al. found that the expression of IGF2BP3 is associated with adverse pathological features such as vascular invasion, neural invasion, and lymph node metastasis [76].

In summary, IGF2BP3 is also a tumor-promoting factor of gastric cancer, participates in the progression of gastric cancer through multiple pathways, and is expected to be a target for inhibiting the progression of gastric cancer (Figure 1).

### 3.7. Colorectal Cancer

Colorectal cancer (CRC) is the third most common cancer and the second leading cause of cancer-related death in the world [77].

In CRC, IGF2BP3 also acts as a tumor promoter. Yang et al. found that IGF2BP3 is significantly overexpressed in colon cancer tissues. IGF2BP3 regulates the cell cycle and angiogenesis by targeting *CCND1* mRNA and *VEGF* mRNA via reading m6A modifications, thus promoting tumor growth in vivo [78]. Zhang et al. confirmed that IGF2BP3 regulates the MEK1/ERK signaling pathway by stabilizing *MEKK1* mRNA in colorectal cancer, and promotes CRC cell proliferation, migration, and invasion. In addition, IGF2BP3 affects *MEKK1* by directly binding to its 3′-UTR [79]. Zhang et al. reported that hsa_cir_0000231 is upregulated in CRC specimens and targets CCND2 to regulate the cell cycle of CRC cells. IGF2BP3 acts by preventing RNA degradation of hsa_circ_0000231 and *CCND2* in SW480 and SW620 cells [80]. Li et al. found that IGF2BP3 synergized with ELAVL1 to regulate mRNA stabilization of *KRAS*, *MAP2K1*, *TPR*, and *CCNH* in CRC [81]. Desi et al. found that LARP1 is upregulated in CRC and may play a role in promoting tumor progression by inhibiting apoptosis. Furthermore, IGF2BP3 and YBX1 are LARP1-interacting proteins and regulate MYC expression and CRC progression [82].

The above studies show that IGF2BP3 has a tumor-promoting effect in CRC. Therefore, targeting IGF2BP3 may be a strategy to inhibit the progression of CRC. Zhang et al. reported that berberine inhibited the PI3K/AKT pathway by downregulating IGF2BP3, thereby inhibiting CRC cell proliferation and cycle transition [83]. Fu et al. found that avenanthramide A (AVN A) can target the miR-129-3p/IGF2BP3 axis in colon cancer, thereby inhibiting tumor proliferation [84]. Therefore, drugs targeting IGF2BP3 may be an approach to inhibit the progression of CRC.

Likewise, IGF2BP3 is involved in constructing prognostic models of colorectal cancer. Liang et al. screened and identified five RNA-binding protein-related prognostic signatures from TCGA (*IGF2BP3*, *PABPC1L*, *PPARGC1A*, *PTRH1*, and *TDRD7*) [85]. Busuioc et al. found that the epithelial–mesenchymal transition gene signature can predict the overall survival rate of colon adenocarcinoma patients (*NOX4*, *IGF2BP3*, *DACT3*, *EEF1A2*, *BMP5*, *GCNT2*, and *SFRP1*) [86]. In 2012, Lochhead et al. confirmed that IGF2BP3 can be used alone as a diagnostic or prognostic biomarker of colorectal cancer [87]. Recently, Glibo et al. further found that IGF2BP3 is an independent prognostic factor of clinical stage II rectal cancer [88].

**Figure 2:**
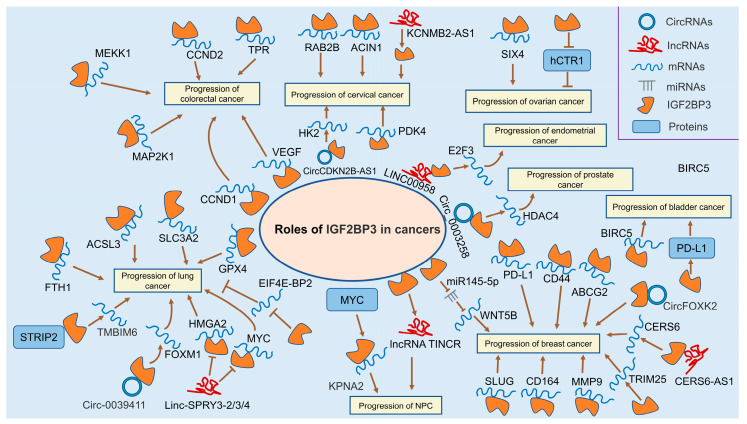
Roles of IGF2BP3 in colorectal, lung, nasopharyngeal, breast, cervical, ovarian, endometrial, prostate, and bladder cancers.

In brief, IGF2BP3 also acts as a tumor-promoting factor in CRC, and the regulation of IGF2BP3 expression may serve as a strategy to inhibit CRC progression (Figure 2).

### 3.8. Oral Cancer

Oral cancer is a major global health problem with increasing incidence among young people [89].

In oral squamous cell carcinoma (OSCC), Liu et al. found that circIGHG is overexpressed in OSCC tissue and cells. circIGHG regulates IGF2BP3 expression through sponging miR-142-5p, thereby promoting OSCC cell proliferation, migration, and invasion [90]. Cui et al. found that circFOXK2 promotes OSCC progression by synergizing with IGF2BP3 to maintain the stability of *GLUT1* mRNA in an m6A-dependent manner [91]. Hwang reported that IGF2BP3 plays an important role in maintaining the stability of *PDPN* mRNA by binding to its 3′UTR, thus promoting CRC invasion in vitro and in vivo [92].

In addition, Tarsitano et al. demonstrated that the expressions of IGF2BP3 and laminin-5 in preoperative biopsy materials are associated with perineural invasion and can be used for accurate preoperative risk stratification in patients with OSCC, suggesting that this strategy can be used to optimize the treatment of people with OSCC [93]. Clauditz et al. found that IGF2BP3 is an independent predictive marker of oral squamous cell carcinoma [94].

In tongue squamous cell carcinoma (TSCC), Wu et al. found that LINC00460 sponges miR-320b and regulates IGF2BP3. The LINC00460/miR-320b/IGF2BP3 axis promotes the growth, migration, and invasion of TSCC cells [95].

Therefore, combining the finding of these several studies shows that IGF2BP3 plays a tumor-promoting role. However, more studies are needed to reveal the mechanisms of IGF2BP3 in oral cancer (Figure 1).

### 3.9. Esophageal Cancer

According to GLOBOCAN 2020, esophageal cancer is the tenth most common malignancy [96].

In esophageal squamous cell carcinoma (ESCC), Huang et al. found that Linc01305 stabilizes *HTR3A* mRNA by interacting with IGF2BP2 and IGF2BP3 to promote proliferation and metastasis [97].

Furthermore, Qian et al. found that IGF2BP3 inhibits the radiosensitivity of esophageal cancer cells by affecting the mRNA stability of *KIF18A* [98]. This study revealed that IGF2BP3 may play a role in the radiosensitivity of tumor cells; more researchers in the future can explore the role of IGF2BP3 in radiosensitization.

IGF2BP3 also appears to be involved in immunity in esophageal cancer. Kono et al. found that the immune responses induced by three HLA-A24-binding peptides (IGF2BP3, LY6K, TTK) improved the prognosis in patients with advanced esophageal squamous cell carcinoma [99]. Zhao et al. used bioinformatics to find that m6A regulators containing IGF2BP3 are associated with the expression of immune molecules and the level of immune infiltration in esophageal cancer [100].

Similarly, IGF2BP3 is used for constructing prognostic models of esophageal cancer. Guo et al. used the TCGA database to construct an m6A methylation-related prognostic signature (*HNRNPC*, *RBM15*, *IGF2BP3*, *METTL16*, and *KIAA1429*). This model can accurately predict the prognosis of patients with esophageal squamous cell carcinoma [101]. Furthermore, Wakita et al. found that high expression levels of IGF2BP3 were significantly associated with poorer prognosis in patients who underwent surgery alone [102].

In summary, IGF2BP3 plays a tumor-promoting role in esophageal cancer and is related to radiosensitivity and the immune response. Therefore, IGF2BP3 may become a powerful marker to inhibit the progression of esophageal cancer (Figure 1).

### 3.10. Lung Cancer

Lung cancer remains the leading cause of cancer death in the world [96].

In lung cancer, IGF2BP3 acts as a tumor-promoting factor. Xu et al. found that IGF2BP3 promotes lung cancer progression by maintaining the mRNA stability of anti-ferroptotic factors in an m6A-dependent manner (*GPX4*, *SLC3A2*, *ACSL3*, and *FTH1*) [103]. Zhang et al. found that STRIP2 is elevated in the non-small cell lung cancer database and clinical samples. The STRIP2–IGF2BP3 axis stimulates the m6A modification of *TMBIM6* mRNA and enhances its stability, thereby promoting tumor growth and metastasis [104]. Lv et al. reported that circ-0039411 enhances the stability of *FOXM1* mRNA by recruiting IGF2BP3 and forms a positive feedback loop. This positive feedback loop promoted LUAD cell proliferation and invasion in vitro and tumor growth and metastasis in vivo [105]. In addition, Brownmiller et al. confirmed that linc-SPRY3-2/3/4 decreases the half-life of IGF2BP3-binding mRNA (*HMGA2* and *MYC*), thereby affecting the apoptosis response [106]. Mizutani et al. found that IGF2BP3 facilitates the activity of the proto-oncogene protein eIF4E through the destabilization of *EIF4E-BP2* mRNA, thus enhancing cell proliferation [107].

The above studies suggest that IGF2BP3 plays a tumor-promoting role in lung cancer. Therefore, drugs that modulate the expression of IGF2BP3 may be a strategy to inhibit the progression of lung cancer. Cui et al. demonstrated that in non-small cell lung cancer, IGF2BP3 can m6A-dependently enhance *TWIST1* stability. Additionally, isoliquiritigenin produces an antitumor effect via the IGF2BP3/*TWIST1* axis [108].

Furthermore, Tomita et al. found that immunogenic peptides from IGF2BP3 effectively kill cancer cells naturally expressing IGF2BP3 through specific cytotoxic T lymphocytes [109]. This suggests that peptides derived from IGF2BP3 are attractive targets for lung cancer immunotherapy.

IGF2BP3 is also involved in constructing different predictive models of lung cancer. Zhang et al. identified a predictive model of m6A regulators for chemotherapy benefit and validated the model in two other cohorts (*ZCCHC4*, *IGF2BP3*, *ALKBH5*, *YTHDF3*, *METTL5*, *G3BP1*, and *RBMX*). The m6A score has a higher predictive power for chemotherapy benefit than other clinicopathological parameters [110]. Zhang et al. constructed a predictive tool for chemotherapy and immunotherapy responses based on five m6A regulators (*G3BP1*, *METTL5*, *ALKBH5*, *IGF2BP3*, and *RBM15B*). Patients with low scores experienced an increased survival benefit with adjuvant chemotherapy and were more sensitive to cancer immunotherapy [111]. Wang et al. constructed a lung cancer prognostic model using platinum resistance-related genes from the TCGA database (*ALDOA*, *CASP12*, *CASP14*, *EXO1*, *FAT1*, *FEN1*, *GDF15*, *HOXB7*, *KRT18*, *MAL*, *MSX1*, *NT5E*, *NTSR1*, *SIX1*, *TXNRD1*, *UBE2S*, and *WFDC2*). The Nomogram plot showed that prediction strongly correlated with actual survival [112].

In conclusion, IGF2BP3 plays a tumor-promoting role in lung cancer, may be related to immunotherapy, and is involved in constructing many prognostic models. It has potential as a therapeutic target for lung cancer (Figure 2).

### 3.11. Nasopharyngeal Cancer

Nasopharyngeal carcinoma (NPC) is a rare malignancy in most parts of the world. However, a significantly higher incidence has long been observed among Cantonese people in southern China [113].

Based on the results of RNA sequencing and bioinformatics analysis, researchers identified *IGF2BP3* as a potential regulator of NPC. Du et al. confirmed that MYC-activated IGF2BP3 promotes NPC cell proliferation and metastasis by influencing the stability of m6A-modified *KPNA2* [114]. Xu et al. found that the expression level of IGF2BP3 is increased in nasopharyngeal carcinoma tissues and cells. IGF2BP3 promotes the migration and invasion of nasopharyngeal carcinoma cells through the AKT/mTOR signaling pathway [115].

Cisplatin-based chemotherapy effectively improves the control of distant metastases in NPC [116]. However, some patients experience treatment failure due to drug resistance. IGF2BP3 was found to be involved in the mechanism of NPC resistance to cisplatin. Zheng et al. reported that lncRNA TINCR is upregulated in nasopharyngeal carcinoma and correlates with poor prognosis. IGF2BP3 interacts with lncRNA TINCR and slows its RNA decay. The IGF2BP3–lncRNA TINCR–ACLY–PADI1–MAPK–MMP2/9 axis plays an important role in nasopharyngeal carcinoma progression and chemoresistance [117].

In summary, IGF2BP3 plays a promoting role in NPC and reduces the sensitivity of NPC cells to cisplatin therapy. Future studies can focus on targeting IGF2BP3 to inhibit NPC progression (Figure 2).

### 3.12. Breast Cancer

According to GLOBOCAN 2020, breast cancer (BC) has become the tumor with the highest incidence rate [96].

IGF2BP3 is a factor promoting many kinds of tumors and plays a cancer-promoting role in breast cancer. IGF2BP3 expression is increased in breast cancer stem cells, which contributes to the self-renewal of tumors. IGF2BP3 was found to target *SLUG* mRNA to prevent decay, thereby participating in the regulation of breast cancer progression. In addition, the binding site is the 5′UTR of the *SLUG* mRNA [118]. Samanta et al. confirmed that IGF2BP3 regulates *MMP9* and *CD164* mRNA and promotes the migration and invasion of breast cancer cells [119]. IGF2BP3 stabilizes *WNT5B* mRNA by inhibiting the expression of miR145-5p, thereby promoting breast cancer progression [120]. IGF2BP3 and miR-3614-3p can competitively bind *TRIM25* mRNA, thus protecting *TRIM25* mRNA from degradation and promoting breast cancer cell proliferation [121]. Bao et al. identified the overexpression of lncRNA CERS6-AS1 in BC tissues and cells. lncRNA CERS6-AS1 promotes the stability of *CERS6* mRNA by combining with IGF2BP3, thereby promoting breast cancer cell proliferation and inhibiting cell apoptosis [122]. Zhang et al. found that CircFOXK2 promotes breast cancer metastasis by regulating IGF2BP3 and miR-370. Moreover, miR-370 can be transferred through exosomes, thereby affecting the metastatic ability of adjacent cells [123]. Kim et al. found that IGF2BP2 and IGF2BP3 promote the metastasis of triple-negative breast cancer (TNBC) by inhibiting the expression of miRNA-200a and degrading *PR* mRNA [124]. Afzali et al. found that IGF2BP3 in breast cancer can be used as a central differentially expressed gene in circRNA networks through bioinformatics analysis [125]. Scott et al. found that seven women diagnosed with BRCA1-like breast cancer had decreased levels of methylation in the *IGF2BP3* promoter region. This suggests that methylation may regulate the expression of IGF2BP3 in some breast cancer patients [126].

Doxorubicin and mitoxantrone are chemotherapeutic drugs for BC, but long-term use produces drug resistance, which affects the survival of patients [127,128]. Samanta et al. demonstrated that the depletion of *IGF2BP3* reduces the expression of ABCG2 and increases drug sensitivity [129].

The role of IGF2BP3 was also confirmed in paclitaxel. Liu et al. reported that IGF2BP3 binds to *CD44* mRNA and enhances the expression of CD44, thereby increasing the IGF2 level of fibroblasts, the proliferation of breast cancer cells, and the drug resistance of paclitaxel [130].

Lint et al. found that the expression level of IGF2BP3 can be used as a potential predictor of sensitivity to PI3K/IGF1R inhibitors [131].

IGF2BP3 is also involved in the immune escape of breast cancer cells. Abnormally high PD-L1 expression on tumor cells mediates the suppression of T-cell activation and tumor immune escape [132]. Wan et al. showed that METTL3 can increase the stabilization of *PD-L1* mRNA in an m6A-IGF2BP3-dependent manner. The knockdown of METTL3/IGF2BP3 can enhance T cell-mediated antitumor immunity by downregulating PD-L1 expression, thus inhibiting the progression of breast cancer [133].

Sjekloča et al. confirmed that the disease-free survival and OS of people with TNBC who were IGF2BP3-positive were significantly shortened [134]. In addition, Ohashi et al. showed that IGF2BP3 expression can be used as an indicator of chemosensitivity in TNBC [135].

In summary, IGF2BP3 promotes tumor progression through multiple pathways in breast cancer, including increased chemotherapeutic drug resistance and tumor immune escape. These findings suggest that IGF2BP3 will be a powerful target for the suppression of breast cancer (Figure 2).

### 3.13. Cervical Cancer

A total of 604,127 new cases and 341,831 deaths were estimated to be related to cervical cancer (CC) in the world in 2020 [96].

Researchers have identified IGF2BP3 as a tumor-promoting factor in cervical cancer. Hu et al. found that METTL3 is upregulated in cervical cancer tissues. Additionally, IGF2BP3 mediates METTL3 to regulate the stability of *RAB2B* mRNA, thus promoting the proliferation of cervical cancer cells [136]. Zhu et al. found that DARS-AS1 positively regulates the expression of IGF2BP3, promotes cervical cancer cell proliferation and invasion, and inhibits apoptosis [137]. Su et al. found that the silencing of METTL3 retards CC cell growth and migration by weakening *ACIN1* mRNA stability via an m6A-IGF2BP3-dependent mechanism [138]. Zhang et al. showed that the levels of lncRNA KCNMB2-AS1 are significantly upregulated in CC tissues, and it sponges miR-130b-5p and miR-4294 in CC cells, which in turn leads to the increased expression of IGF2BP3. IGF2BP3 can stabilize lncRNA KCNMB2-AS1 through binding KCNMB2-AS1 through three m6A modification sites on KCNMB2-AS1. Therefore, these biomolecules can form a positive regulatory loop. The inhibition of the lncRNA KCNMB2-AS1/miR-130b-5p/miR-4294/IGF2BP3 axis could retard tumor growth in vivo [139]. Researchers found that circCDKN2B-AS1 cooperated with IGF2BP3 to regulate the stability of *HK2* mRNA in cervical cancer, thereby promoting aerobic glycolysis and cancer progression [140].

In addition, researchers also found that IGF2BP3 is involved in doxorubicin resistance in cervical cancer. Li et al. demonstrated that IGF2BP3 is involved in m6A-regulated *PDK4* mRNA stability, thereby regulating cervical cancer cell proliferation, glycolysis, and sensitivity to doxorubicin treatment [141].

Taken together, IGF2BP3, as a key molecule in multiple mechanisms, plays a tumor-promoting role in cervical cancer. Studies on IGF2BP3 have provided new insights into cervical cancer treatment (Figure 2).

### 3.14. Ovarian Cancer

According to relevant reports, ovarian cancer is the leading cause of gynecological cancer death in the United States and the fifth most common cause of cancer death in American women [142]. Epithelial ovarian cancer accounts for the majority of malignant ovarian tumors.

IGF2BP3 increases the stability of *SIX4* mRNA, thus promoting ovarian cancer cell proliferation, migration, and invasion, as well as tube formation [143].

Platinum-based chemotherapy is the first-line treatment for ovarian cancer [144]. Hsu et al. identified that the decreased expression of IGF2BP3 and Lin28B reduces ovarian cancer cell proliferation, migration, and invasion and increases platinum sensitivity by increasing hCTR1 protein expression [145].

Wiedemeyer et al. found that a biomarker panel (p53, p16, and IGF2BP3) can be used to predict the potential need for adjuvant therapy in patients with stage I ovarian cancer [146]. This may facilitate comprehensive treatment decisions in patients with ovarian cancer.

Overall, the above studies show that IGF2BP3 plays a role in tumor promotion in ovarian cancer. In addition, IGF2BP3 affects the occurrence and development of ovarian cancer through other pathways that need additional study (Figure 2).

### 3.15. Endometrial Cancer

Endometrial cancer (EC) is a common gynecologic malignancy in developed countries, and its incidence is increasing worldwide [147]. Visser et al. showed that IGF2BP3 has diagnostic value in endometrial cancer and is associated with aggressive features such as deep myometrial invasion and lymphovascular space invasion. Furthermore, IGF2BP3 with L1CAM is the best combination to distinguish low- from high-grade endometrial cancer [148]. Wang et al. found that IGF2BP3 is upregulated in EC tissues and is associated with a poorer prognosis. In addition, LINC00958 acts as an oncogene to assist IGF2BP3 in stabilizing the mRNA of *E2F3*, thereby promoting EC progression [149] (Figure 2).

### 3.16. Prostate Cancer

Prostatic cancer (PCa) is a common malignancy among men in developed countries and is the second leading cause of cancer-related death in the United States [150].

Less research has been conducted on how IGF2BP3 affects prostate cancer progression. In PCa, hsa_circ_0003258 binds to IGF2BP3 and enhances the stability of *HDAC4* mRNA, thus activating the ERK signaling pathway. Moreover, the results of methylated RNA immunoprecipitation analysis showed that *HDAC4* is highly enriched in m6A precipitation. Additionally, hsa_circ_0003258/IGF2BP3/HDAC4 and hsa_circ_0003258/miR-653-5p/ARHGAP5 crosstalk can promote prostate cancer progression [151].

Szarvas et al. found elevated expression levels of IGF2BP3 in serum and tissue samples from PCa patients compared with those of non-PCa patients [152]. This suggests that IGF2BP3 is promising as a non-invasive diagnostic marker of prostate cancer.

The role of IGF2BP3 in PCa is gradually being revealed, but its mechanism of action in prostate cancer needs further study (Figure 2).

### 3.17. Kidney Cancer

According to the literature, in 2020, there were 431,288 new cases and 179,368 deaths related to kidney cancer worldwide [96].

IGF2BP3 plays a tumor-promoting role in kidney cancer. Gu et al. confirmed that lncRNA DMDRMR cooperates with IGF2BP3 to stabilize their targets (*CDK4*, *COL6A1*, *LAMA5*, and *FN1*) in an m6A-dependent manner to promote the proliferation, migration, and invasion of renal cancer cells [153]. Xie et al. found that IGF2BP3 regulates the stability of lncRNA CDKN2B-AS1 in renal clear cell carcinoma, thereby promoting the expression of NUF2. The IGF2BP3/lncRNA CDKN2B-AS1/NUF2 axis promotes renal cell carcinoma cell growth and metastasis in vitro and in vivo [154]. Furthermore, Tschirdewahn et al. demonstrated that high plasma levels of IGF2BP3 are an independent predictor of disease-specific survival in renal cell carcinoma [155]. This may clinically optimize risk stratification and treatment strategies for patients with renal cell carcinoma.

### 3.18. Bladder Cancer

Bladder cancer is one of the most common cancers in the world. An estimated 573,278 new cases of bladder cancer were diagnosed in 2020 [96].

Sitnikova et al. first showed that IGF2BP3 is an independent prognostic marker in bladder urothelial carcinoma [156]. Huang et al. found that IGF2BP3 promotes the proliferation, migration, and invasion of bladder cancer cells by activating the JAK/STAT signaling pathway [157].

IGF2BP3 plays an important role in the promotion of bladder cancer progression through fine particulate matter (PM2.5). Liu et al. elucidated that PM2.5 upregulates the expression of METTL3 through promoter hypomethylation and transcription factor HIF1A. Furthermore, PM2.5-induced m6A modification regulates the mRNA stability of *BIRC5* through METTL3/IGF2BP3, thus promoting the proliferation and metastasis of bladder cancer [158].

IGF2BP3 also participates in the construction of prediction models for bladder cancer. Cui et al. developed bladder cancer prognostic models using m6A regulators (*IGF2BP3*, *LRPPRC*, *YTHDC1*, *YTHDF2*, and *WTAP*). The risk score of the model is related to tumor grade and tumor stage. Furthermore, they confirmed that IGF2BP3 positively regulates the expression of PD-L1 in bladder cancer [159].

Taken together, the findings of the above studies suggest that IGF2BP3 is a tumor-promoting factor in bladder cancer and can serve as a prognostic marker of bladder cancer. In addition, researchers have revealed the regulatory relationship between IGF2BP3 and PD-L1 in bladder cancer. Studies in the future can focus on the role of IGF2BP3 in immune regulation (Figure 2).

## 4. Discussion

Malignant tumors are the main cause of premature death in humans and pose a serious threat to human health [160]. Gene mutations, epigenetic changes, and the activation of signaling pathways in organisms play important roles in tumor occurrence and progression. *IGF2BP3* was originally reported as a highly expressed gene in pancreatic cancer. In subsequent studies, its biological functions were gradually revealed, including promoting the embryonic development of organisms, regulating fetal megakaryocytes, and inducing fetal hemoglobin in adult erythroblasts. With the development of m6A technology, IGF2BP3 was found to be a part of the m6A-binding protein and to participate in m6A modification together with YTHDF1, YTHDC2, and other binding proteins. In acute myelogenous leukemia, IGF2BP3 was observed to enhance the stability of *RCC2* mRNA by reading m6A modification sites, thereby promoting cancer progression. Similarly, IGF2BP3 was found to suppress ferroptosis in HCC cells by promoting the stability of *NRF2* mRNA in an mA-dependent manner. In gastric cancer, IGF2BP3 directly recognizes and binds to the m6A site on *HDGF* mRNA and enhances its stability. In glioma, IGF2BP3 enhances the stability of lncRNA WEE2-AS1 in an mA-dependent manner, attenuating drug sensitivity. These findings suggest that IGF2BP3, as an m6A-binding protein, plays a crucial role in tumor progression. In addition, IGF2BP3 can promote tumor progression by regulating the mRNA stability of downstream targets through other pathways, such as through an RISC-dependent mechanism, competing with miRNAs for the common binding site on the 3′-UTR of targets, and affecting miRNA production to indirectly regulate the targets. These possible regulatory pathways provide ideas for future explorations of the specific mechanisms of action of IGF2BP3.

IGF2BP3 is associated with chemotherapy resistance in cervical cancer by regulating *PDK4* mRNA stability. Research findings have revealed the potential of targeting IGF2BP3 to prevent chemotherapy resistance. During glucose metabolism, IGF2BP3 can maintain the stability of *GLUT1* mRNA, which is related to aerobic glycolysis in oral squamous cell carcinoma. Researchers may develop strategies based on this metabolic phenomenon. In addition, the knockdown of METTL3/IGF2BP3 can enhance antitumor immunity by downregulating PD-L1 expression. These results suggest that targeting IGF2BP3 may have new implications for tumor immunotherapy. Because IGF2BP3 is almost absent in normal tissues other than in reproductive tissues, it may be an important target for cancer therapy. Although no inhibitors directly targeting IGF2BP3 have been found, METTL3 methyltransferase inhibitors and FTO demethylation inhibitors have been produced. STM2457 is a highly potent and selective METTL3 inhibitor, which has been tested in acute myeloid leukemia [161]. N-CDPCB was found to be an FTO inhibitor [162]. In addition, the phytochemicals isocorydine derivative and isoliquiritigenin inhibit the expression of IGF2BP3 to repress tumor cell proliferation. These findings suggest that IGF2BP3-targeted inhibitors may be developed in the future. The expression of IGF2BP3 is increased in many tumors, such as hepatocellular carcinoma, colorectal cancer, lung cancer, and breast cancer, and is associated with poor prognosis, indicating that IGF2BP3 may be a biomarker of malignant tumors. Researchers have used bioinformatics techniques to develop multiple prognostic models that include the *IGF2BP3* gene. These provide evaluation indicators for the assessment of patient condition.

In conclusion, the dysregulation of IGF2BP3 plays a crucial role in tumor development and progression by affecting different features of human cancers, including tumor cell proliferation, migration, metastasis, chemotherapy resistance, radiosensitivity, and immune response (We summarized roles and targets of IGF2BP3 in various human cancers in Table S1; Full names of the genes mentioned in the text in Table S2). m6A modification has gradually become the focus of cancer research, but the role of IGF2BP3 in this process is still poorly understood. We must deeply study the mechanism of action of IGF2BP3 and strive to develop its potential in targeted tumor therapy. In addition, research results need to be translated into clinical practice to truly improve the prognosis of cancer patients.

## Data Availability

Not applicable.

## Supplementary Materials

The supporting information can be downloaded at: https://www.mdpi.com/article/10.3390/ijms24119423/s1.
