# Supplementary material for: Targeting IGF2BP3 in Cancer
Source: Int J Mol Sci. 2023 May 29;24(11):9423. doi: 10.3390/ijms24119423 (PMC10253605; doi:10.3390/ijms24119423)
Supplement: Supplementary file 1 [file ijms-24-09423-s001.zip › ijms-2304500-supplementary.pdf]

Table S1. Roles and targets of IGF2BP3 in various human cancers.

Tumor type	Target	Validated m6A modification	Function	References
Leukemia	RCC2	√	Inhibits the apoptosis	[28]
	COX-2		Inhibits the apoptosis	[29]
Glioma	ZNRF3		Promotes the proliferation and TMZ resistance and inhibits apoptosis	[39]
	LncRNA WEE2-AS1	√	Promotes the proliferation, invasion and dasatinib resistance	[42]
Ewing sarcoma	ABCF1		Promotes the growth and migration	[50]
	CD164		Promotes the migration	[51]
	IGF1R		Promotes the migration and growth.	[52]
Hepatocellular carcinoma	TRAF5		Promotes the proliferation and metastasis	[55]
	Lnc-CTHCC	√	Promotes the proliferation, invasion and angiogenesis	[56]
	NRF2	√	Inhibits the ferroptosis and promotes sorafenib resistance	[62]
Gastric cancer	PKMYT1	√	Promotes the invasion and migration	[68]
	HDGF	√	Promotes the growth, metastasis and angiogenesis	[69]
	SLC7A5		Promotes the proliferation	[70]
	MYC		Promotes the proliferation and migration	[71]
	CD44		Promotes the migration, invasion and EMT	[72]
Colorectal cancer	CCND1	√	Promotes the proliferation and regulates cell cycle	[78]
	VEGF	√	Promotes angiogenesis	[78]
	MEKK1		Promotes the proliferation, migration, and invasion	[79]
	CCND2		Promotes the proliferation and regulates cell cycle	[80]
	MAP2K1		Promotes the proliferation	[81]
	TPR		Promotes the proliferation	[81]
Oral cancer	GLUT1	√	Promotes aerobic glycolysis	[91]
	PDPN		Promotes the growth and invasion	[92]
Esophageal cancer	HTR3A		Promotes the proliferation and migration	[97]
	KIF18A		Promotes the proliferation, migration, invasion and radiosensitivity	[98]
Lung cancer	GPX4	√	Inhibits the ferroptosis	[103]
	SLC3A2	√	Inhibits the ferroptosis	[103]
	ACSL3	√	Inhibits the ferroptosis	[103]
	FTH1	√	Inhibits the ferroptosis	[103]
	TMBIM6	√	Promotes the proliferation, migration, invasion and inhibits apoptosis	[104]
	FOXM1		Promotes the proliferation, migration and EMT	[105]
	MYC		Inhibits the apoptosis	[106]
	HMGA2		Inhibits the apoptosis	[106]
	EIF4E-BP2		Promotes the proliferation	[107]
	TWIST1	√	Promotes the proliferation, migration and invasion	[108]

Nasopharyngeal carcinoma	KPNA2	√	Promotes the proliferation, invasion, migration and EMT	[114]
	LncRNA TINCR		Promotes the proliferation, metastasis, and cisplatin resistances	[117]
Breast cancer	SLUG		Promotes self-renewal and tumor initiation	[118]
	MMP9		Promotes the migration and invasion	[119]
	CD164		Promotes the migration and invasion	[119]
	MiR145-5p		Activates Hippo signaling	[120]
	TRIM25		Promotes the proliferation and regulates cell cycle	[121]
	CERS6		Promotes the proliferation and inhibits apoptosis	[122]
	PR		Promotes the migration and invasion	[124]
	ABCG2		Promotes doxorubicin and mitoxantrone resistance	[129]
	CD44		Promotes the proliferation and paclitaxel resistance	[130]
	PD-L1	√	Promotes immune escape	[133]
Cervical cancer	RAB2B		Promotes the proliferation	[136]
	ACIN1	√	Promotes the proliferation and migration	[138]
	KCNMB2-AS1	√	Promotes the proliferation and inhibits the apoptosis	[139]
	HK2		Promotes the aerobic glycolysis	[140]
	PDK4	√	Promotes the proliferation, glycolysis, and doxorubicin resistance	[141]
Ovarian cancer	SIX4		Promotes the proliferation, migration and invasion, and tube formation	[143]
	hCTR1		Promotes the proliferation, migration and invasion and platinum resistance	[145]
Endometrial cancer	E2F3		Promotes the proliferation, migration, and invasion	[149]
Prostate cancer	HDAC4	√	Promotes EMT and metastasis	[151]
Kidney cancer	CDK4	√	Promotes the proliferation	[153]
	FN1	√	Promotes the invasion and metastasis	[153]
	COL6A1	√	NA	[153]
	LAMA5	√	NA	[153]
	CDKN2B-AS1		Promotes the growth and metastasis	[154]
Bladder cancer	BIRC5	√	Promotes the proliferation and metastasis	[158]
	PD-L1		NA	[159]

Table S2. Full names of the genes mentioned in the text

Abbreviation	Full name
IGF2BP3	insulin-like growth factor 2 mRNA-binding protein 3
TRAF5	tumor necrosis factor receptor-associated factor 5
EIF4E-BP2	eukaryotic translation initiation factor 4E binding protein 2
RCC2	regulator of chromosome condensation 2
IL-18	interleukin 18
HuR	human antigen R
COX-2	cyclooxygenase 2
ZNRF3	zinc and ring finger 3
ABCF1	ATP binding cassette subfamily F member 1
CXCR4	C-X-C motif chemokine receptor 4

NRF2	NFE2 like BZIP transcription factor 2
YBX1	Y-box binding protein 1
ZC3H13	zinc finger CCCH-type containing 13
YTHDF1	YTH N6-methyladenosine RNA binding protein F1
TRMT10C	tRNA methyltransferase 10C, mitochondrial RNase P subunit
YTHDF2	YTH N6-methyladenosine RNA binding protein F2
RRP8	ribosomal RNA processing 8
TRMT6	tRNA methyltransferase 6 non-catalytic subunit
LRPPRC	leucine rich pentatricopeptide repeat containing
G6PD	glucose-6-phosphate dehydrogenase
ZNF239	zinc finger protein 239
BIRC5	baculoviral IAP repeat containing 5
FOXM1	forkhead box M1
CENPA	centromere protein A
KIF4A	kinesin family member 4A
DTYMK	deoxythymidylate kinase
PRC1	protein regulator of cytokinesis 1
KIF2C	kinesin family member 2C
TRIP13	thyroid hormone receptor interactor 13
THYM	thymoma
TPX2	TPX2 microtubule nucleation factor
PKMYT1	protein kinase, membrane associated tyrosine/threonine 1
HDGF	heparin binding growth factor
SLC7A5	solute carrier family 7 member 5
MYC	MYC proto-oncogene, bHLH transcription factor
ZEB1	zinc finger E-box binding homeobox 1
SNAI1	snail family transcriptional repressor 1
CCND1	cyclin D1
VEGF	vascular endothelial growth factor
MEKK1	mitogen-activated protein kinase kinase kinase 1
CCND2	cyclin D2
ELAVL1	ELAV like RNA binding protein 1
KRAS	KRAS proto-oncogene, GTPase
MAP2K1	mitogen-activated protein kinase kinase 1
TPR	translocated promoter region, nuclear basket protein
CCNH	cyclin H
LARP1	La ribonucleoprotein 1, translational regulator
YBX1	Y-box binding protein 1
PABPC1L	poly(A) binding protein cytoplasmic 1 like
PPARGC1A	PPARG coactivator 1 alpha
PTRH1	peptidyl-tRNA hydrolase 1 homolog
TDRD7	tudor domain containing 7
NOX4	NADPH oxidase 4
DACT3	dishevelled binding antagonist of beta catenin 3

EEF1A2	eukaryotic translation elongation factor 1 alpha 2
BMP5	bone morphogenetic protein 5
GCNT2	glucosaminyl (N-acetyl) transferase 2 (I blood group)
SFRP1	secreted frizzled related protein 1
GLUT1	glucose transporter 1
PDPN	podoplanin
HTR3A	5-hydroxytryptamine receptor 3A
KIF18A	kinesin family member 18A
LY6K	lymphocyte antigen 6 family member K
TTK	TTK protein kinase
HNRNPC	heterogeneous nuclear ribonucleoprotein C
RBM15	RNA binding motif protein 15
METTL16	methyltransferase 16, N6-methyladenosine
KIAA1429	vir like m6A methyltransferase associated
GPX4	glutathione peroxidase 4
SLC3A2	solute carrier family 3 member 2
ACSL3	acyl-CoA synthetase long chain family member 3
FTH1	ferritin heavy chain 1
STRIP2	striatin interacting protein 2
TMBIM6	transmembrane BAX inhibitor motif containing 6
HMGA2	high mobility group AT-hook 2
TWIST1	twist family bHLH transcription factor 1
ZCCHC4	zinc finger CCHC-type containing 4
ALKBH5	alkB homolog 5, RNA demethylase
YTHDF3	YTH N6-methyladenosine RNA binding protein F3
METTL5	methyltransferase 5, N6-adenosine
G3BP1	G3BP stress granule assembly factor 1
RBMX	RNA binding motif protein X-linked
RBM15B	RNA binding motif protein 15B
ALDOA	aldolase, fructose-bisphosphate A
CASP12	caspase 12
CASP14	caspase 14
EXO1	exonuclease 1
FAT1	FAT atypical cadherin 1
FEN1	flap structure-specific endonuclease 1
GDF15	growth differentiation factor 15
HOXB7	homeobox B7
KRT18	keratin 18
MAL	mal, T cell differentiation protein
MSX1	msh homeobox 1
NT5E	5'-nucleotidase ecto
NTSR1	neurotensin receptor 1
SIX1	SIX homeobox 1
TXNRD1	thioredoxin reductase 1

UBE2S	ubiquitin conjugating enzyme E2 S
WFDC2	WAP four-disulfide core domain 2
KPNA2	karyopherin subunit alpha 2
ACLY	ATP citrate lyase
PADI1	peptidyl arginine deiminase 1
MMP2	matrix metallopeptidase 2
MMP9	matrix metallopeptidase 9
SLUG	snail family transcriptional repressor 2
WNT5B	Wnt family member 5B
TRIM25	tripartite motif containing 25
CERS6	ceramide synthase 6
PR	progesterone receptor
ABCG2	ATP binding cassette subfamily G member 2
IGF2	insulin like growth factor 2
PD-L1	programmed cell death 1 ligand 1
RAB2B	RAB2B, member RAS oncogene family
ACIN1	apoptotic chromatin condensation inducer 1
HK2	hexokinase 2
PDK4	pyruvate dehydrogenase kinase 4
SIX4	SIX homeobox 4
L1CAM	L1 cell adhesion molecule
E2F3	E2F transcription factor 3
HDAC4	histone deacetylase 4
ARHGAP5	Rho GTPase activating protein 5
CDK4	cyclin dependent kinase 4
COL6A1	collagen type VI alpha 1 chain
LAMA5	laminin subunit alpha 5
FN1	fibronectin 1
NUF2	NUF2 component of NDC80 kinetochore complex
METTL3	methyltransferase 3, N6-adenosine-methyltransferase complex catalytic subunit
HIF1A	hypoxia inducible factor 1 subunit alpha
BIRC5	baculoviral IAP repeat containing 5
LRPPRC	leucine rich pentatricopeptide repeat containing
YTHDC1	YTH N6-methyladenosine RNA binding protein C1
WTAP	WT1 associated protein
IGF1R	insulin like growth factor 1 receptor
